# Sulfonate-Based Triazine Multiple-Electron Anolyte for Aqueous Organic Flow Batteries

**DOI:** 10.1021/acsami.3c05850

**Published:** 2023-07-25

**Authors:** Juan Asenjo-Pascual, Cedrik Wiberg, Mahsa Shahsavan, Ivan Salmeron-Sanchez, Pablo Mauleon, Juan Ramon Aviles Moreno, Pilar Ocon, Pekka Peljo

**Affiliations:** † Department of Applied Physical Chemistry, Universidad Autónoma de Madrid, c/Fco. Tomás y Valiente 7, Cantoblanco, Madrid 28049, Spain; ‡ Department of Organic Chemistry, Universidad Autónoma de Madrid, Madrid 28049, Spain; § Research Group of Battery Materials and Technologies, Department of Mechanical and Materials Engineering, Faculty of Technology, University of Turku, Turku 20014, Finland

**Keywords:** triazine, anolyte, multiple-electron storage, aqueous organic electrolyte, redox flow battery, energy storage

## Abstract

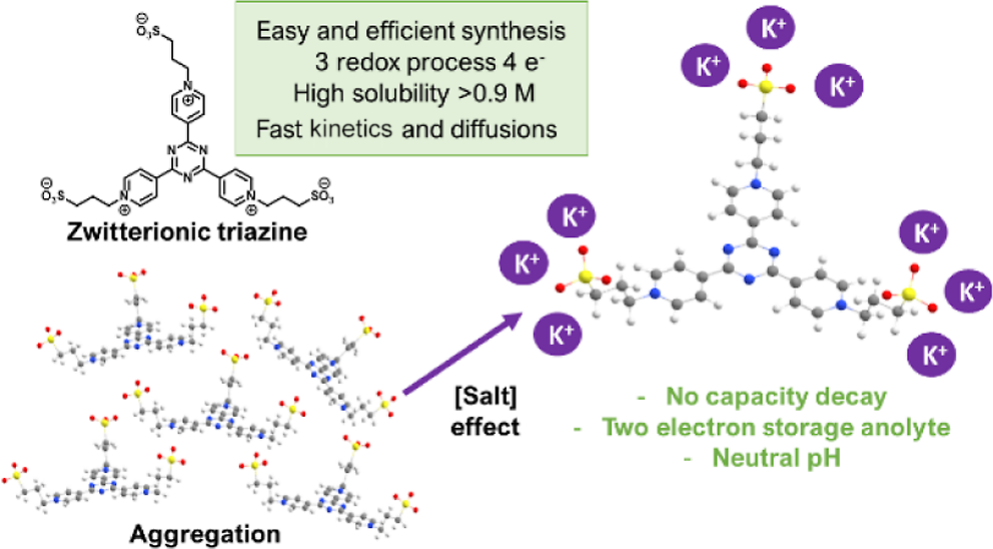

A new highly soluble triazine derivative **(SPr)**_**3**_**4TpyTz** showing three reversible redox processes with fast kinetics and high diffusion coefficients has been synthesized using an efficient, low-cost, and straightforward synthetic route. Concentrated single cell tests and DFT studies reveal a tendency of the reduced triazine species to form aggregates which could be avoided by tuning the supporting electrolyte concentration. Under the right conditions, **(SPr)**_**3**_**4TpyTz** shows no capacity decay and good Coulombic, voltage, and energy efficiencies for the storage of two electrons. The storage of further electrons leads to a higher capacity decay and an increase of the electrolyte pH, suggesting the irreversible protonation of the generated species. So, a plausible mechanism has been proposed. A higher concentration of **(SPr)**_**3**_**4TpyTz** shows slightly higher capacity decay and lower efficiencies due to the aggregate formation.

## Introduction

Currently, renewable energies, such as solar or wind, have decreased their production costs, making them more competitive compared to fossil sources. However, a frequent intermittency of solar and wind power must be solved before their implementation into the electrical grid on the large scale.^1,2^ In this sense, the energy storage devices emerge as a great candidate to solve this issue. Specifically, one of the most studied strategies are flow batteries (FBs) which could enable large-scale energy storage in a safe way at low cost. Moreover, flow batteries show very high flexibility as their energy and power capability can be tailored for specific needs, unlike for Li-ion batteries where capacity and power are coupled.^3^ Among the different FBs studied, vanadium flow batteries are being commercialized,^4^ but many alternative nonaqueous and aqueous FBs have also been proposed.^5^ Aqueous organic FBs (AOFBs) are one of the most promising candidates due to the use of earth-abundant-based materials in their electrolytes, higher safety, potentially low cost, and most importantly, the tunability of the redox active materials as redox potential, solubility, among other characteristics.^6−8^

Because of the expectations put in AOFBs, many organic and inorganic molecules have been studied as potential redox active molecules. Different families of electrolytes have been studied as for example: quinones,^9,10^ TEMPO-derivatives,^11^ viologens,^12^ ferrocene derivatives,^13^ polypeptides,^14^ among others. Strategies like the addition of electron-withdrawing or -donating groups to tune the redox potential or the addition of hydrophilic groups to increase the solubility have been developed during last years.^15−17^

Many example molecules reported in the literature are able to store just one electron excluding quinones and derivatives thereof where the redox reactions commonly involve two electrons and, in some circumstances and depending on pH, two protons.^18−21^ The main challenge of the multiple electron storage concerns the chemical stability of the generated species in multiple different oxidation states. Just a few examples where the organic molecule enables the storage of two electrons in aqueous systems are described in the literature.^22−24^ The preparation of a bigger RAM (redox active material) could avoid one of the major problems of RFBs, the crossover. Larger molecules are less prone to pass through the pores and channels of the ion exchange membranes, thus leading to more stable systems. Liang et al. reported a new triazine-based anolyte which can store up to three electrons.^25^ The planar structure of the triazine benefits from electronic delocalization, stabilizing the radicals generated in the reduction of its pyridine moieties. One of the main problems of this derivative is the long synthesis proposed with four steps of counterion exchange to increase the solubility.

In this work, we propose a new zwitterionic triazine derivative, which can easily be synthetized in multigram scale by a two-step synthesis with high atom economy. Herein, we report a new anolyte which is able to electrochemically reversibly transfer four electrons in three redox processes. This molecule was tested in a flow battery cell showing promising results, especially almost no capacity fade was observed in the right conditions. Furthermore, DFT calculations have shown how the reduced species can interact forming aggregates and decreasing the solubility.

## Results and Discussion

**Figure 1:**
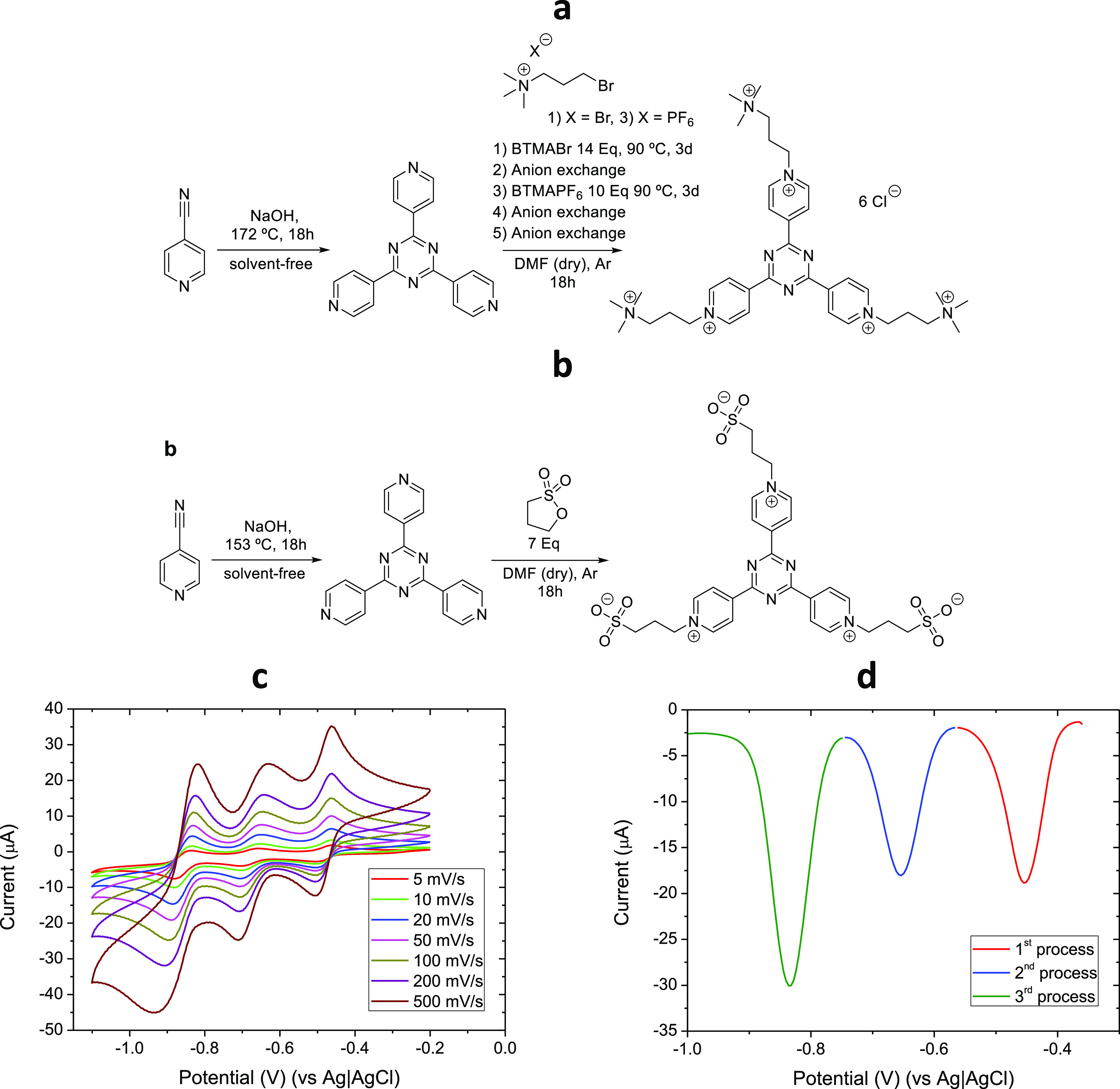
(a) Synthesis proposed for the first triazine-based anolyte.^25^ (b) Synthesis of **(SPr)**_**3**_**4TpyTz**. (c) CV at different scan rates and (d) DPV using 1 mM of **(SPr)**_**3**_**4TpyTz** in 1 M KCl. Working electrode: glassy carbon, reference electrode: Ag|AgCl (3 M KCl); and counter electrode: Pt wire.

A new anolyte for FBs based on a variant of the trispyridinium-triazine molecule reported by Liang et al. (Figure 1a)^25^ was synthetized. The synthesis approach depicted in Figure 1b has promise to simplify and decrease the preparation cost of triazine anolyte by avoiding the anion exchange steps by obtaining the zwitterionic product, **(SPr)**_**3**_**4pyTz**.

The prepared compound exhibits three reversible redox processes at −0.47, −0.62, and −0.82 V versus Ag|AgCl (3 M KCl) (Figure 1c). In the cyclic voltammetry (CV) measurement, the peak separation of the first process is scan rate independent, but peak separation for the second and third process increases with the increase in scan rate. Therefore, the first process can be considered reversible, while the other two are quasi-reversible. The differential pulse voltammogram (DPV) (Figure 1d) shows three reduction peaks where the two redox processes at less negative potentials correspond to single-electron reduction. By contrast, at more negative potential, the peak of the DPV has double the current, indicating two-electron reduction, as shown in Figure 1d. The diffusion coefficients and the kinetic rate constants of each reversible process were studied using linear sweep voltammetry (LSV) on a glassy carbon rotating disk electrode (RDE) (Table 1 and Figures S7–S12). As expected based on CVs, the first process has faster kinetics than the two others. As the fast kinetics and high diffusion coefficients are matched with the high solubility 0.94 in 1 M KCl (determined by UV/vis, Figure S13), initial evaluation of the molecule is promising and the performance should be tested in single-cell. Note that third redox process presents significantly lower diffusion coefficient which could be ascribed to the aggregate formation.

**Table 1:** Redox Potential, Kinetic Constants, and Diffusion Coefficient for Each Redox Process

	*E* (V) *vs* Ag|AgCl (3 M KCl)	*k* (cm/s) × 10^–^^3^	*D* (cm^2^/s) × 10^–^^6^
**1**st **process**	–0.47	19.60	1.95
**2**nd **process**	–0.62	2.32	1.53
**3**rd **process**	–0.82	0.16	0.56

**Figure 2:**
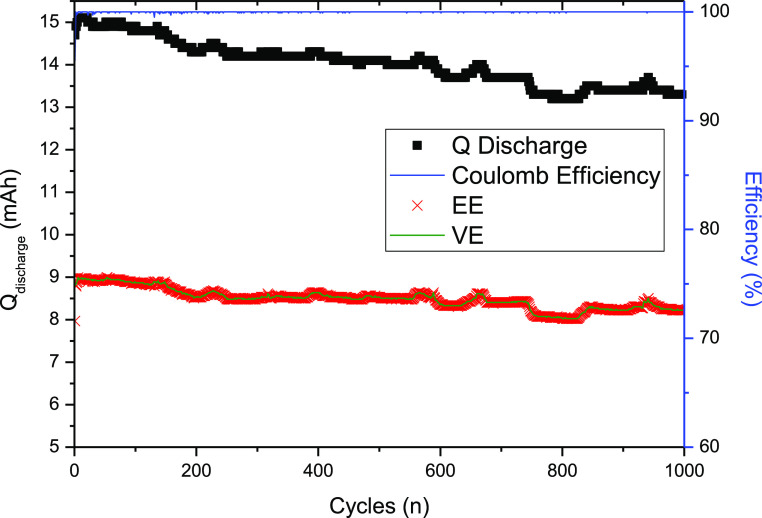
Galvanostatic cycling by using both electrons of **(SPr)**_**3**_**4TpyTz** 25 mM in 1 M KCl and 100 mM K_4_[Fe(CN)_6_] in 1 M KCl at 60 mA/cm^2^ for 1000 cycles. Discharge capacity, Coulombic, voltage, and energy efficiencies evolution.

The promising electrochemical properties encouraged testing the performance of **(SPr)**_**3**_**4TpyTz** in a single cell. To demonstrate the performance of this new anolyte, a neutral-pH flow battery was assembled using 25 mM of **(SPr)**_**3**_**4TpyTz** versus 125 mM K_4_[Fe(CN)_6_] in 1 M KCl as positive electrolyte using Nafion 212 as an ion exchange membrane. The battery was cycled galvanostatic at 60 mA/cm^2^ at room temperature for 1000 cycles (11.7 days) with the cutoff potentials selected to access first and second reduction of the **(SPr)**_**3**_**4TpyTz**. The achieved capacity of *ca*. 15 mA h corresponds to 74.6% of the theoretical capacity (20.1 mA h for two electrons storage). Deviations between the theoretical and the measured capacity are mainly attributed to the system resistance. As depicted in Figure 2, this battery shows a Coulombic efficiency of 100% and voltage and energy efficiencies close to 75% in all cycles, which is comparable to the VRFB systems.^26^ The capacity decay observed during cycling was −0.163 mAh/day which corresponds to −1.08%/day with respect to the maximum capacity reached. The fluctuations in the capacity evolution during cycling could be ascribed to some drops on the vial walls. To the best of our knowledge, this compound represents one of the most stable systems reported in the literature and its synthesis is one of the cheapest and shortest.

After these promising results provided by the battery at low concentrations, battery tests at higher concentrations were performed in the same conditions with 100 mM of **(SPr)**_**3**_**4TpyTz** in 1 M KCl versus 300 mM of K_4_[Fe(CN)_6_] in 1 M KCl. Once the battery started to be charged, after 15 cycles, the flow on the negative side was blocked due to the electrolyte precipitation, preventing further cycling (Figure S14). After that, the electrolytes were analyzed by ^1^H NMR and CV. Both experiments demonstrate no degradation of the redox active material (Figures S15 and S16). Based on these results, it was speculated that the reduced state is not fully soluble, while the battery is charging. By increasing the concentration of the reduced state, at some point, the reduced state precipitates blocking the flow.

**Figure 3:**
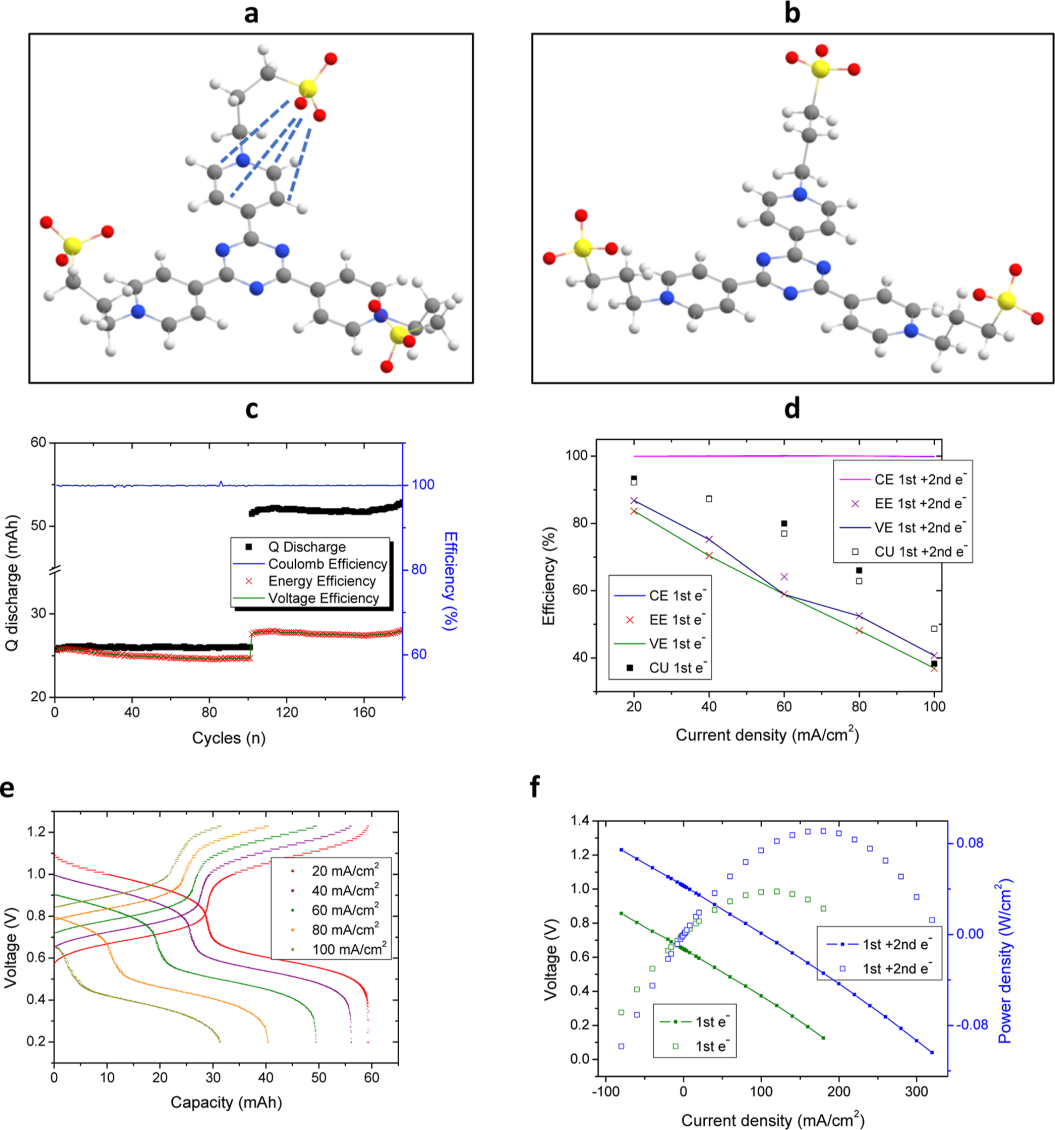
(a,b) Optimized structures of the oxidized and reduced states of **(SPr)**_**3**_**4TpyTz** at the CAM-B3LYP/6-31G** level of theory. (c) Galvanostatic cycling of 100 mM of **(SPr)**_**3**_**4TpyTz** in 3 M KCl vs 100 mM K_4_[Fe(CN)_6_]. Cycling a constant current reaching one (1–100 cyles) and two electrons (101–180 cycles) at 60 mA/cm^2^ using 0.9 and 1.25 V upper cutoffs, respectively, and 0.2 V as lower cutoff. (d) Discharge capacity, Coulombic, voltage, and energy efficiencies evolution. (e) Charge–discharge capacity vs voltage profile at different current densities. (f) Polarization (line) and power density (scatter) curves for one and two electron battery at 50% SOC.

Some density functional theory (DFT) calculations were employed to investigate the structure of the oxidized and reduced form of the **(SPr)**_**3**_**4TpyTz** to deeply understand the effect of solubility while considering electronic considerations (see Figure 3a,b). The geometric structures of both redox states were optimized using the CAM-B3LYP/6-31G** level of theory with empirical dispersion correction (GD3BJ) and the implicit solvent model based on density (SMD). As it can be observed in Figure 3a, the sulfonate group of the oxidized state is intramolecularly interacting with the pyridine of the triazine. This kind of interactions has been observed previously for sulfonated-viologen^27^ and it has been corroborated by NOESY spectra (Figure S4). By contrast, in the reduced form, the sulfonate group is open and “free” to interact intermolecularly with the pyridine core of other molecules. The possibility of this intermolecular interaction and the central symmetry which has been demonstrated that favors the formation of aggregates.^28^ This fact may explain why the solubility of the reduced form can be compromised during charging. It has been reported that zwitterionic species’ solubility can be enhanced by increasing the salt concentration of the environment.^29^ The solubility of the oxidized molecules is apparently not a problem as it was measured to be greater than 900 mM in 1 M KCl. Nevertheless, when the salt is removed, even the oxidized molecules are poorly soluble in water (<100 mM in DI water, Figure S17). All in all, it is evident that the salt is playing a vital role in the solubility of the triazine derivatives. The interaction between the zwitterionic species can lead to the formation of aggregates that becomes insoluble in water. By adding enough salt into the solution, these interactions can be avoided or at least lessened, enhancing the solubility. Furthermore, better solvation of the triazine molecules could lead in higher stability due to the hindered interaction between the molecules. In this sense, a plausible battery may work considering more concentrated supporting electrolyte solution. Thereby, a battery using 3 M KCl solution in the anolyte was tested (Figure 3c–f).

A flow cell with 12 mL of 0.1 M **(SPr)**_**3**_**4TpyTz** in 3 M KCl as the anolyte and 50 mL of 0.1 M K_4_[Fe(CN)_6_] in 1 M KCl as the catholyte [0.2 M of electrons in the capacity limiting side (CLS)] was tested inside a glovebox. First, the cell was studied just analyzing the first electron and then subsequently reach the second and the third electrons. Initially, the cell was galvanostatically charged at 60 mA/cm^2^ with 0.9 and 0.2 V as cutoffs for 100 cycles (Figure 3c, from cycle 1 to 100). The total discharge capacity was 26.11 mA h which corresponds to 81.2% of the capacity utilization for one electron. Moreover, different current densities were tested (20, 40, 60, 80, and 100 mA/cm^2^) (Figure 3e). In all currents, the Coulombic efficiency was near 100%. The voltage and energy efficiencies range from 83.7 (lower current) to 36.9% (highest current). The capacity utilization reached 93.3 to 38.3% from the lowest to the highest current densities, respectively (Figure 3d). All these results show that higher current densities lead to higher Ohmic and mass transport resistances, increasing the overpotential. As the cutoff voltage was kept constant, the achieved SOC decreases with the increase in current density. After analyzing the performance of the battery at different current densities, the system was charged to 50% SOC (with respect to the first electron of the anolyte) and the EIS experiments (Figure S18), and the polarization curves were recorded (Figure S19). From the polarization curve, the resistance of the whole system was calculated as 2.81 Ω·cm^2^. A power density peak of 37.6 mW/cm^2^ was achieved between 100 and 120 mA/cm^2^. The cell was cycled 100 times suggesting a great stability showing no capacity decay over 18 h. After that, the second electron was studied. First, the cell was cycled galvanostatically at the same current (60 mA/cm^2^) but using 1.25 and 0.2 V as cutoffs (Figure 3c from cycle 101 to 180). The total discharge capacity at this current was around 52 mA h which represents 76.9% of the capacity utilization. When cycled at different current densities, the Coulombic efficiency was around 100%, the voltage and energy efficiencies range from 86.8 to 40.7%, and the capacity utilization reached 92.2 (lowest current) and 48.7% (highest current) (Figure 3d). Subsequently, the system was theoretically charged to 50% SOC (with respect to the second electron of the anolyte) and the EIS experiments (Figure S20) and the polarization curves were recorded. A power density peak of 91.1 mW/cm^2^ was achieved at 180 mA/cm^2^. The modest power density would be increased in an optimized cell with a lower Ohmic resistance . The cell was cycled 80 times showing no capacity decay over 29 h. Large multiple-electron storage materials typically have lower diffusion coefficients, but these bigger molecules could avoid the permeability through the ion exchange membrane which is one of the most limiting phenomena in FB performance. The suppression of the crossover and the stability of the proposed triazine derivative place this work as a special redox active material which can work without showing any capacity decay over 3 days.

Finally, the third redox process was studied by increasing the upper cutoff to 1.5 V. This resulted in a high-capacity decay (>1%/cycle) on each cycle, and a significant increase in the pH of the electrolyte was observed (Figure S21 and Table S1). In comparison, no pH change was observed if cycling was limited to 1st or 2nd reduction. This fact is in good agreement with the proposed protonation reaction responsible of the pH change in the third redox process and, and therefore, the observed capacity decay.

**Figure 4:**
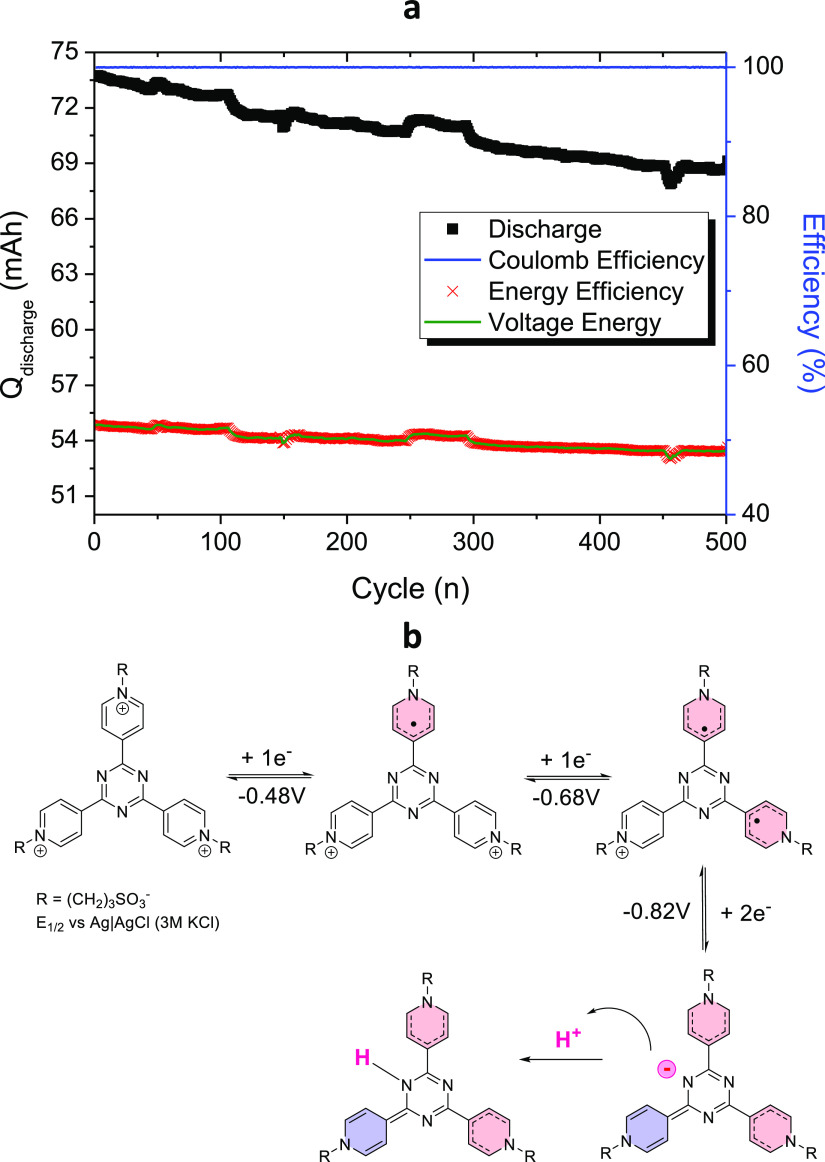
(a) Galvanostatic cycling at 60 mA/cm^2^ of 200 mM of **(SPr)**_**3**_**4TpyTz** in 3 M KCl vs 300 mM K_4_[Fe(CN)_6_] in 1 M KCl. Discharge capacity, Coulombic, voltage, and energy efficiencies evolution. (b) Proposed mechanism for each reduction step.

Finally, battery testing was carried out at a higher concentration using 200 mM **(SPr)**_**3**_**4TpyTz** in 3 M KCl and 300 mM K_4_[Fe(CN)_6_] in 1 M of KCl (0.4 M of electrons in the negative CLS). The battery was galvanostatically cycled for 500 cycles at 60 mA/cm^2^ (14 days). The system shows a capacity decay of 4.47 mA h, corresponding to a capacity retention of 93.8% and a capacity decay of 0.012%/cycle or 0.44%/day (Figure 4a). Both electrolytes were checked by CV ensuring that after 14 days no crossover was observed (Figure S22). Furthermore, impedance before and after cycling show a slightly increase in the resistance (20% higher) probably due to the formation of aggregates and their interaction with the IEM (Figure S23). This capacity decay is ascribed to the precipitation of the triazine probably due to the change in the electrolyte composition because of the crossover of the potassium through the ion exchange membrane. This battery also shows slightly lower energy efficiency probably due to the higher concentration of the triazine leading to lower “free potassium” concentration, decreasing electrolyte conductivity, and increasing the formation of triazine aggregates.

To understand the stable performance of the **(SPr)**_**3**_**4TpyTz**, we can focus on the mechanism of the redox reaction (Figure 4b). The first reduction reduces one of the pyridinium rings leading to a greatly stable radical delocalized throughout the whole structure. Second reduction forms a biradical species which is in correlation with the mechanism proposed by Liang et al.^25^ Finally, the third reduction process generates a negative charge in the N-atom of the triazine which gets protonated making the third process irreversible. This leads to a fast capacity decay observed if the third plateau is reached during charging, as well as a large decrease in the pH of the electrolyte (Figure S21 and Table S1). Triazine reported by Liang et al.^25^ could be reduced three times without significant adverse effects. This is most likely because all three pyridium groups are reduced before the triazine core. In our case, it looks like the reduction of the triazine occurs with the potential range of the reduction of the last pyridinium, leading to high-capacity decay and the dramatic change in the pH after just four cycles.

This work represents one of the unique examples where no capacity decay has been showed in the right composition of electrolyte, albeit at moderate concentrations of 100 mM. The larger triazine based anolyte is stable enough to store two electrons without any kind of degradation probably due to the high delocalization of the generated radicals into the pyridinium and triazine rings. Furthermore, the large size of this triazine derivative avoids crossover through the ion exchange membrane. The optimization of the electrolyte could enable the use of more concentrated solutions which could lead to a higher capacity for this system. To do this, the interaction between the reduced state of **(SPr)**_**3**_**4TpyTz** could be studied by molecular dynamics (MD) in order to understand how to avoid this interaction which precipitate the electrolyte at some SOC. Furthermore, the synthesis of new triazine derivatives where the central symmetry is avoided could avoid the formation of aggregates. This work opens the door to the synthesis of new triazine derivatives which are new candidates for multiple electron storage showing high stability during cycling.

## Conclusions

An efficient and low-cost synthesis of a new triazine derivative have been proposed. The electrochemical characterization of **(SPr)**_**3**_**4TpyTz** shows fast kinetics and diffusions coefficients for the three redox processes. The performance of this new material at different concentrations has been evaluated showing a limiting issue related to the formation of aggregates and therefore a decrease in the solubility of the reduced state. DFT studies have shed light into this issue and by varying the supporting electrolyte concentration this problem has been partially solved. A 100 mM solution (200 mM of electrons) showed no capacity decay after 200 cycles with the optimized electrolyte composition. The mechanism of each redox step as well as an explanation of higher capacity decay in the third redox process has been given. This work may be the first step for future synthesis of new triazine derivatives avoiding the central symmetry as well as the study of new additives to avoid the aggregates formation. These triazine derivatives are very interesting redox active materials due to their capacity to store multiple electrons in neutral pH.

## Experimental Section

4-cyanopyridine, sodium hydroxide, propane sultone, and dimethylformamide (DMF) chlorohydric acid were purchased from Sigma-Aldrich and used without further purification. RDE were conducted using a Metrohm Autolab Motor Controller. Both CV and RDE tests were performed using an Autolab electrochemical system II PGSTAT30 potentiostat. The solubilities were measured using an UV–vis spectrophotometer (PerkinElmer, Lambda 365).

### Synthesis of 2,4,6-Tris-(4-pyridyl)-1,3,5-triazine (**TPT**)

30 g of 4-cyanopyridine (288.2 mmol, 3 equiv) was placed in a 100 mL round-bottom flask and heated to 153 °C. Once the whole solid gets liquid, 0.912 g of powdered NaOH (22.8 mmol, ≈0.1 equiv) was added in small portions. The resulting mixture was stirred 18 h until all the liquid become pale solid. The solid was dissolved using concentrated HCl aqueous solution 50 mL and sonicated for 30 min at room temperature. After that, the solution was neutralized using 6 M aqueous solution of NaOH until pH 7. The white product was purified by redissolving it in 10 mL of concentrated HCl aqueous solution and neutralized using 6 M aqueous solution of NaOH to achieve the desired product. The white powder was cleaned using 3 × 50 mL of acetone and finally with 50 mL of water, achieving 19 g of the pale white solid (60.8 mmol, 63.3%). Spectroscopic data were in good agreement with those reported in the literature [1]. ^1^H NMR (300 MHz, CDCl_3_): δ 8.95 (s, 6H), 8.57 (s, 6H).

### Synthesis of 3,3′,3″-[(1,3,5-Triazine-2,4,6-triyl)tris(pyridine-1-ium-4,1-diyl)]tris(propane-1-sulfonate) **(SPr)**_**3**_**4TpyTz**

2 g of TPT (6.4 mmol, 1 equiv) and 9 g of 1,3-propane sultone (73.7 mmol 4 equiv) were dissolved in 50 mL of DMF and heated to reflux for 18 h in a 250 mL round-bottom flask. After this time, a brown pale solid appears in the bottom of the flask. The solution was cooled until room temperature was reached to ensure the precipitation of all the product. The solid was filtered using a Buchner funnel and washed with cool DMF and acetone. The solid was purified by dissolving it in water and precipitating using MeOH. So, 3.6 g of the pure product (5.3 mmol, 82.8%) was achieved as a brown pale solid. ^1^H NMR (300 MHz, D_2_O): δ 9.40 (d, *J* = 6.2 Hz, 6H), 9.32 (d, *J* = 7.1 Hz 2H), 5.01 (t, *J* = 7.4 Hz, 6H), 3.11 (t, *J* = 7.2 Hz, 6H), 2,63 (p, *J* = 7.2 Hz, 6H) ^13^C NMR (75 MHz, D_2_O): δ 169.5 (s), 149.7 (s), 146.0 (s), 127.5 (s), 60.4 (s), 47.0 (s). 26.2 (s).

NMR spectra and the synthetic routes for both compounds can be found in the Supporting Information.

### Electrochemical Characterization of **(SPr)**_**3**_**4TpyTz**

CVs were recorded in a three-electrode cell using a glassy carbon (GC) disk (diameter of 3.0 mm) as the working electrode (WE), Pt wire as the counter electrode, and Ag|AgCl as the reference electrode (RE). 1 mM **(SPr)**_**3**_**4TpyTz** in 1 M of KCl aqueous solution was tested at different scan rates and room temperature. Formal potentials were estimated by taking the average between the cathodic and anodic peak. The diffusion behavior was confirmed studying the CV at different scan rates from 5 to 500 mV/s, showing a linear pattern by plotting the peak current versus *v*^1/2^.

DPVs were recorded using the same system, and the experimental conditions were 4 mV potential increase, 50 mV amplitude, and 500 ms pulse period. The relationship between the reduction peak intensity of each redox process let us estimate that the third process involves the double of electrons than the first and second processes.

The diffusion coefficient was calculated using the Levich approximation as given in eq S1. Koutecký–Levich analysis (see eq S2) at low overpotentials can be extrapolated to infinite rotation rate and fitted to the Butler–Volmer equation (see eq S3) to get the standard rate constant of the reduction process.

All the equations and analysis used to study the electrochemical properties of **(SPr)**_**3**_**4TpyTz** can be found in the Supporting Information.

### Solubility of **(SPr)**_**3**_**4TpyTz**

The solubility of the **(SPr)**_**3**_**4TpyTz** was measured by UV–vis spectroscopy. Calibration curves were obtained using aqueous solutions at different concentrations. An aliquot of the saturated solution of the corresponding triazine derivative was diluted until the absorption of the sample fits the corresponding calibration curves. UV–vis spectra at different concentrations and calibration curves can be found in the Supporting Information.

### DFT Calculations

Optimized geometries at the CAM-B3LYP/6-31G** level of theory with empirical dispersion correction (GD3BJ) and the implicit solvent model based on density (SMD). The coordinates for both states of charges studied can be found in the Supporting Information.

### Cell Testing

The home-made flow cell with flat flow fields was set up using two composite bipolar plates (carbon-polyolefin), graphite felts electrodes (SGL GFD 4.6 EA, used as received and compressed to 3 mm), two sheets of gasket (expanded, Teflon), and Nafion 212 membrane from Dupont. The active area of the cell was 5 cm^2^. A Chonry BT600M peristaltic pump was calibrated with Masterflex C-Flex tubbing (Cole-Parmer) and used to circulate the electrolyte through the system at a flow rate of 60 mL/min. The reservoirs and the cell were place inside a glovebox purged with nitrogen (MBRAUN). After circulating the electrolyte for 30 min, the initial resistance of the system was determined using impedance spectroscopy with a BioLogic SP-300 potentiostat. For battery measurements, a LANHE Battery Tester 400 W was used. The cell was galvanostatically charged/discharged at room temperature in different voltage ranges (each cutoff can be found in the footnote of each figure) at different current densities (20, 40, 60, 80, and 100 mA/cm^2^, 5 cycles) and cycled for several cycles at 60 mA/cm^2^. The polarization curves were measured point-by-point by applying a current of +1 mA/cm^2^ during 10, 10 s resting, and 10 s at −1 mA/cm^2^, so the SOC is expected to do not change during the experiment.

Extra information about the different batteries and the analysis of the electrolytes before and after cycling are shown in the Supporting Information.

## Supporting Information Available

Information regarding the synthesis of **(SPr)**_**3**_**4TpyTz** and its chemical and electrochemical characterization, solubility determination, optimized geometries used in DFT calculations, cell-test details, and brief comparison between **(SPr)**_**3**_**4TpyTz** and other organic electrolytes (PDF)
