# Supplementary material for: Sulfonate-Based Triazine Multiple-Electron Anolyte for Aqueous Organic Flow Batteries
Source: ACS Appl Mater Interfaces. 2023 Jul 25;15(30):36242–9. doi: 10.1021/acsami.3c05850 (PMC10401562; doi:10.1021/acsami.3c05850)
Supplement: Supplementary file 1 — am3c05850_si_001.pdf [file am3c05850_si_001.pdf]

Supporting Information

Sulfonate-based triazine multiple-electron anolyte for Aqueous Organic Flow Batteries.

Juan Asenjo-Pascual,[a,b,*] Cedrik Wiberg,[c] Mahsa Shahsavan,[c] Ivan Salmeron-Sanchez,[a] Juan Ramon Aviles Moreno,[a] P. Mauleón,[b] and P. Ocón,[a] and Pekka Peljo.[c,*]

[a] Department of Applied Physical Chemistry, Universidad Autónoma de Madrid, c/Fco. Tomás y Valiente 7, Cantoblanco, 28049 Madrid, Spain. [b] Department of Organic Chemistry, Universidad Autónoma de Madrid.

[c] Research Group of Battery Materials and Technologies, Department of Mechanical and Materials Engineering, Faculty of Technology, University of Turku, 20014 Turku, Finland.

Corresponding author: juan.asenjo@uam.es and pekka.peljo@utu.fi

Table of contents:

1. Synthesis and characterization

2. Electrochemical characterisation

3. Solubility. UV-Vis

4. DFT Calculation

5. Cell-tests

 i) Cell stuck

 ii) CV + 1H NMR after stuck

 iii) solubility with different KCl concentrations

 iv) PEIS 0 and 50 % SOC

 v) Load-Curve

 vi) Third electron

 vii) CV after cycling

6. Comparison with other organic electrolytes

1. Synthesis and characterization

Synthesis of 2,4,6-tris-(4-pyridyl)-1,3,5-triazine TPT[1]

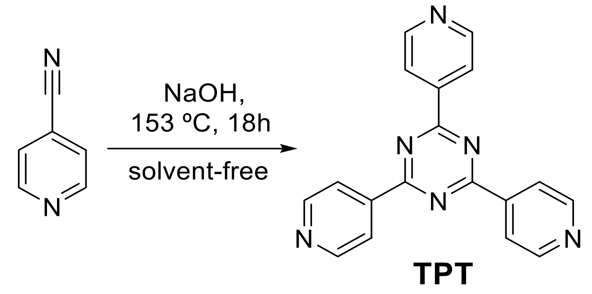

Scheme 1: Synthetic route towards TPT.

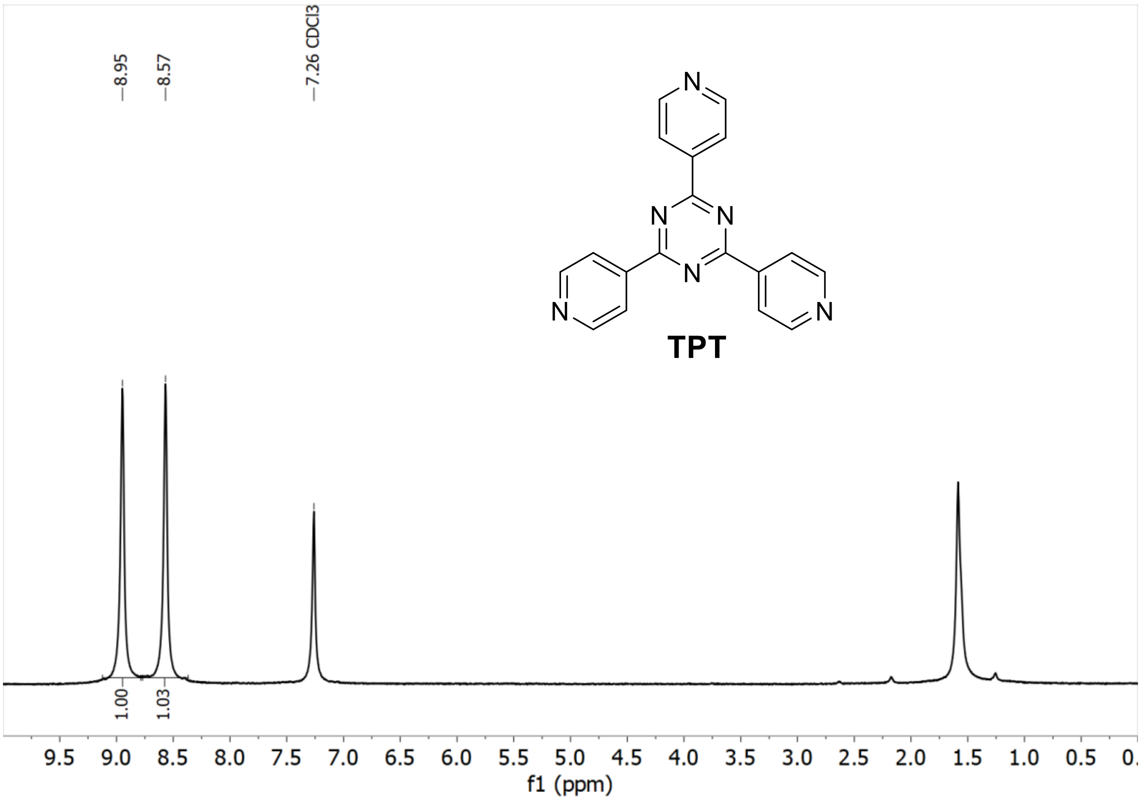

Figure S1: ^{1}H NMR in $CDCl_3$ of compound TPT.

Synthesis of 3,3',3''-((1,3,5-triazine-2,4,6-triyl)tris(pyridine-1-ium-4,1-diyl))tris(propane-1-sulfonate) (SPr)34TpyTz

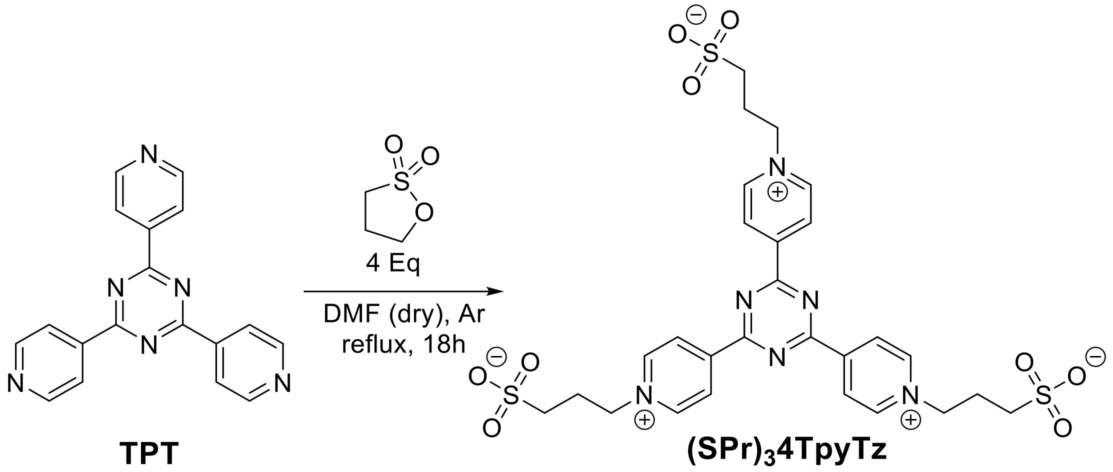

Scheme 2: Synthetic route towards **(SPr)34TpyTz**.

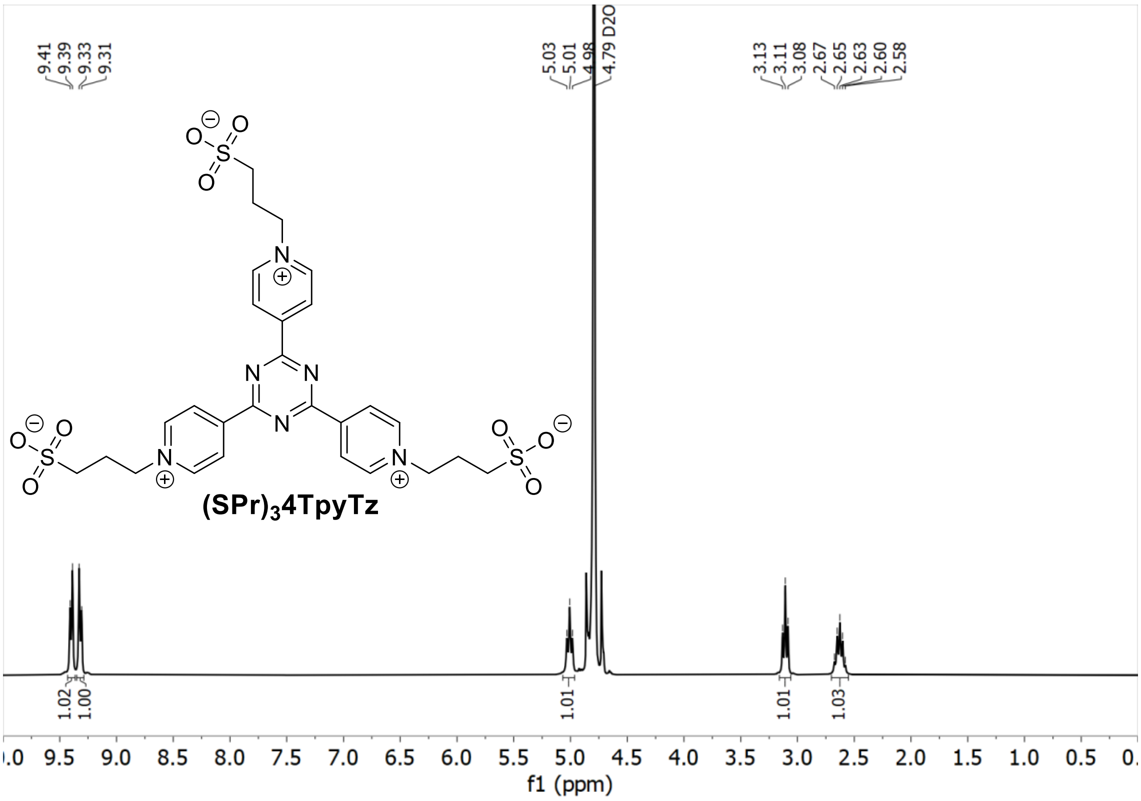

Figure S2: ^{1}H NMR in D_2O of compound **(SPr)34TpyTz.**

2. Electrochemical characterization of $(SPr)_3$4TpyTz

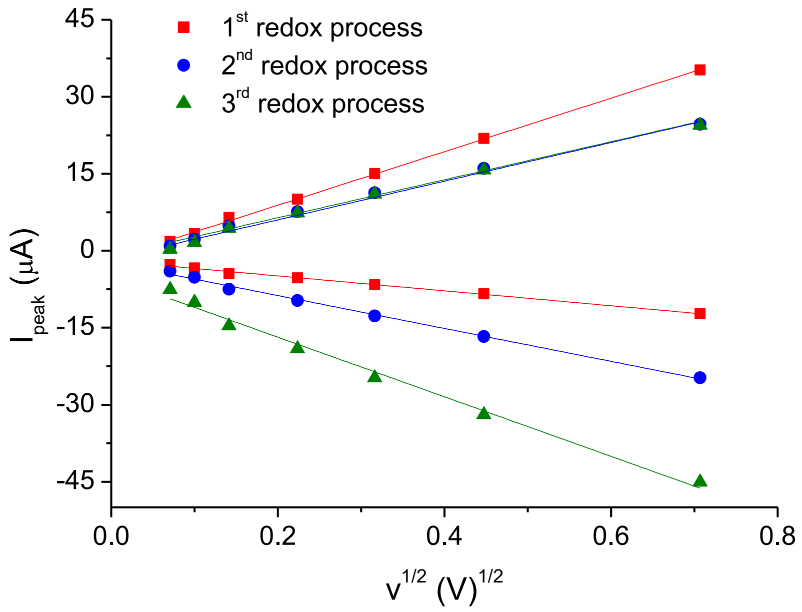

Figure S5: Peak current (anodic and cathodic) vs square root of the scan rate for the first (red) second (blue) and third (green) redox processes of 1mM **$(SPr)_3$4TpyTz** in 1 M KCl.

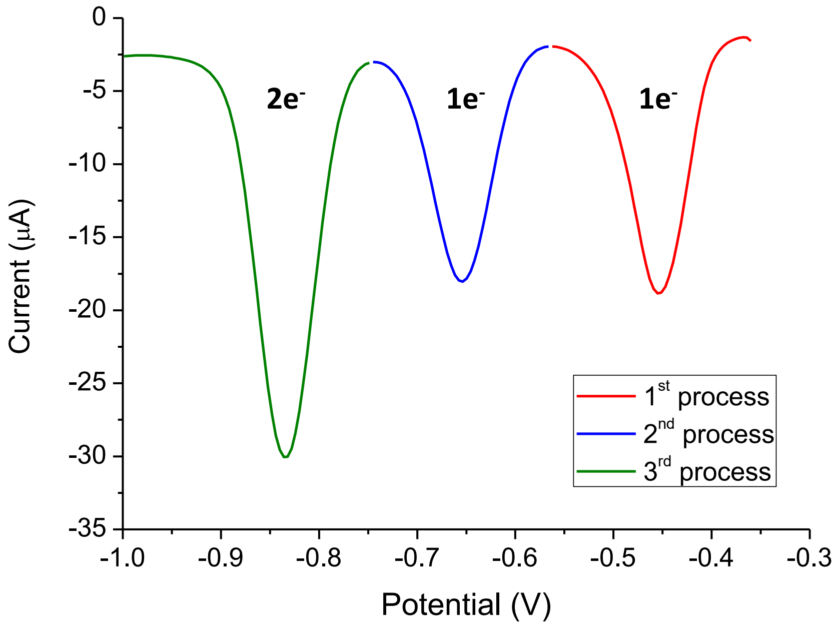

Figure S6: Differential Pulse Voltammetry of 1mM **$(SPr)_3$4TpyTz** in 1 M KCl.

Rotating Disk Electrode (RDE) measurements and Kinetic analysis

The diffusion coefficient was calculated using the Levich approximation as given in Eq. S1. Koutecký-Levich analysis (see Eq. S2) at low overpotentials can be extrapolated to infinite rotation rate and fitted to the Butler-Volmer equation (see Eq. S3) to get the standard rate constant of the reduction process.

$$I_L = 0.620nFAC_oD^{2/3}v^{-1/6}\omega^{1/2} \quad \textbf{(S1)}$$

Where I_L is the limiting current (A), n is the number of electrons involved in the redox reaction and in all the cases n = 1 (the difference between the reduction and oxidation peaks studied correspond to values of 59 mV or even higher), F is the Faraday constant (C/mol), A is the electrode area (cm^2), C_o is the molar concentration of the redox active material (mol/cm^3), D is the diffusion coefficient (cm^2/s), υ is the kinematic viscosity (cm^2/s) and ω the angular rotation rate of the electrode (rad/s).

$$\frac{1}{i} = \frac{1}{i_k} + \frac{1}{0.620nFAD^{2/3}v^{-1/6}\omega^{1/2}C_o} \quad \textbf{(S2)}$$

$$\log i_K = \log nFC_oAk_o + \frac{\alpha nF\eta}{2.303RT} \quad \textbf{(S3)}$$

Where I_K is the kinetic current (A), C_o is the molar concentration of the redox active material (mol/cm^3), α is the transfer coefficient, k_o is the kinetic constant (cm/s), η is the overpotential |E-E$_{1/2}$| (V), R is the gas constant (J/mol·K) and T the temperature (K).

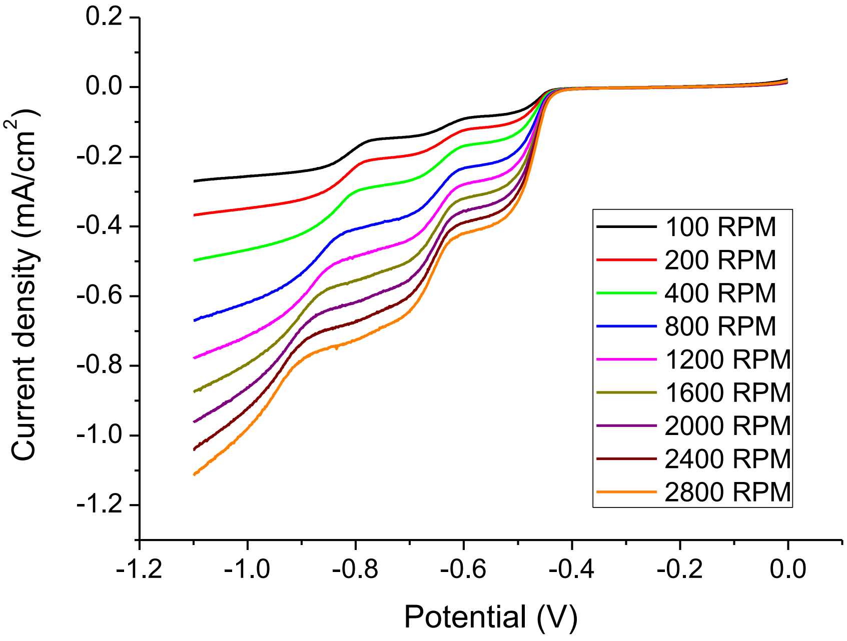

Figure S7: Rotating Disk Electrode study of the reduction of 1 mM of **(SPr)$_3$4TpyTz** in 1 M KCl on a GC electrode at rotation rates from 100 to 2800 rpm.

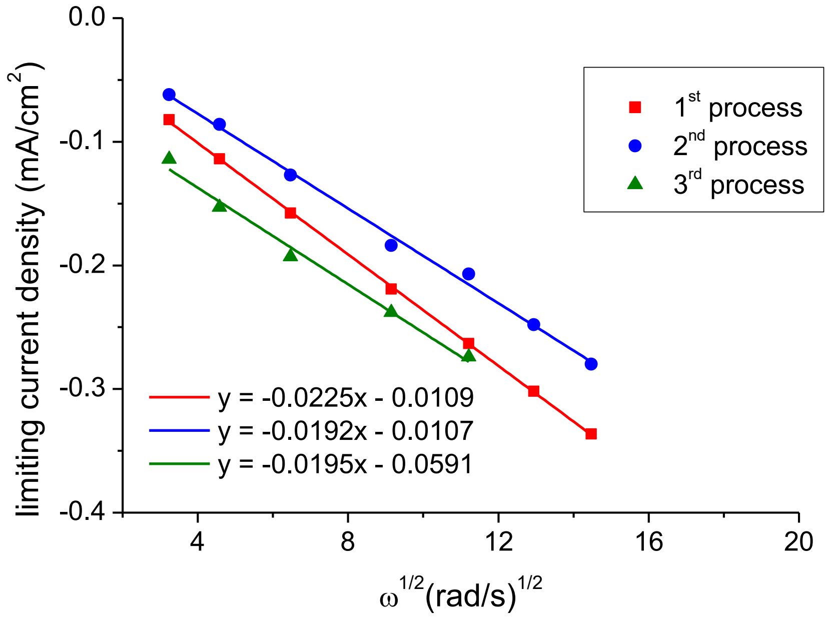

Figure S8: Levich plot for the first (red), second (blue), and third (green) redox processes (limiting current *vs* square root of rotation rate in rad/s) of 1 mM of **(SPr)$_3$4TpyTz** in 1 M KCl. Note that all the slopes are almost equal but the fact that the third process involves 2 electrons make the diffusion coefficient lower.

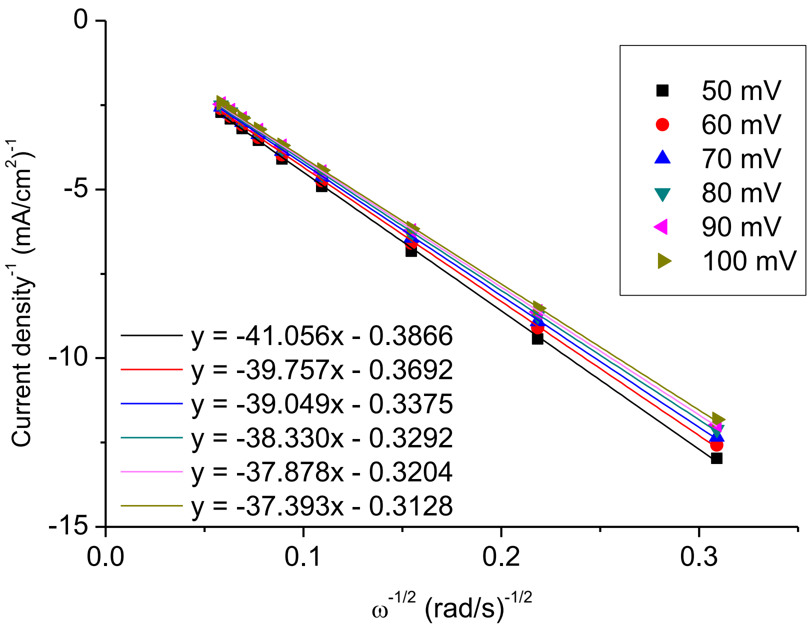

Figure S9: Koutecky-Levich plot at different overpotentials for the first reduction process of 1 mM of **(SPr)$_3$4TpyTz** in 1 M KCl.

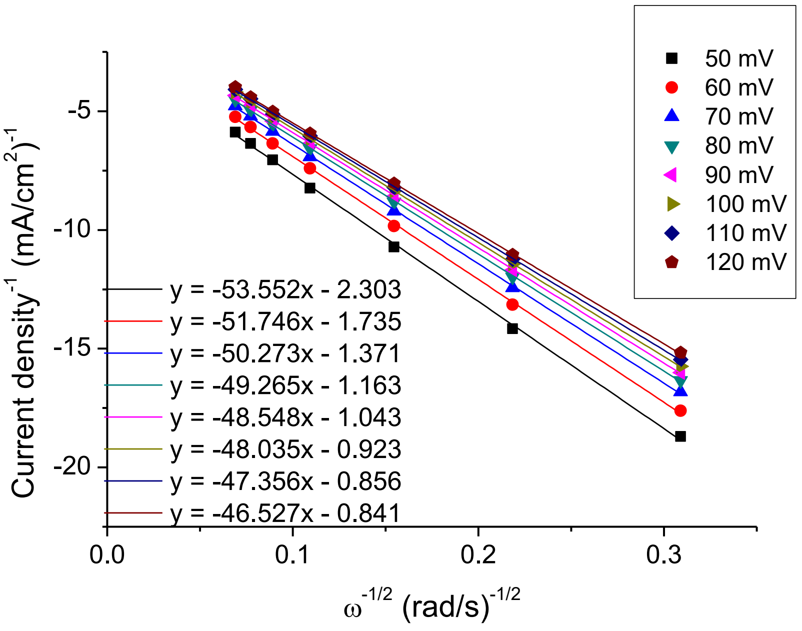

Figure S10: Koutecky-Levich plot at different overpotentials for the second reduction process of 1 mM of **(SPr)$_3$4TpyTz** in 1 M KCl.

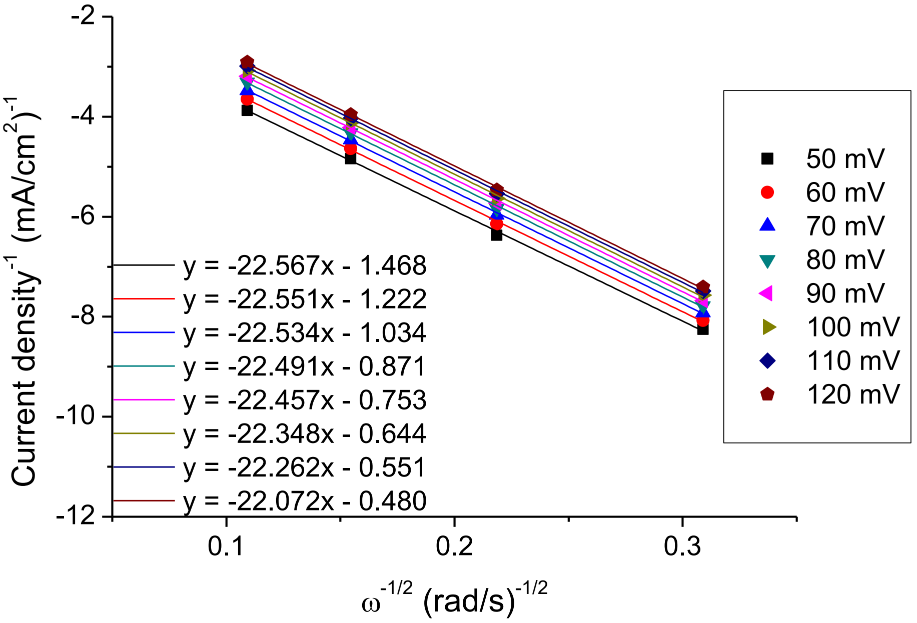

Figure S11: Koutecky-Levich plot at different overpotentials for the third reduction process of 1 mM of **(SPr)$_3$4TpyTz** in 1 M KCl. The third process present contribution of the other two processes moving away from the Koutecky-Levich approach.

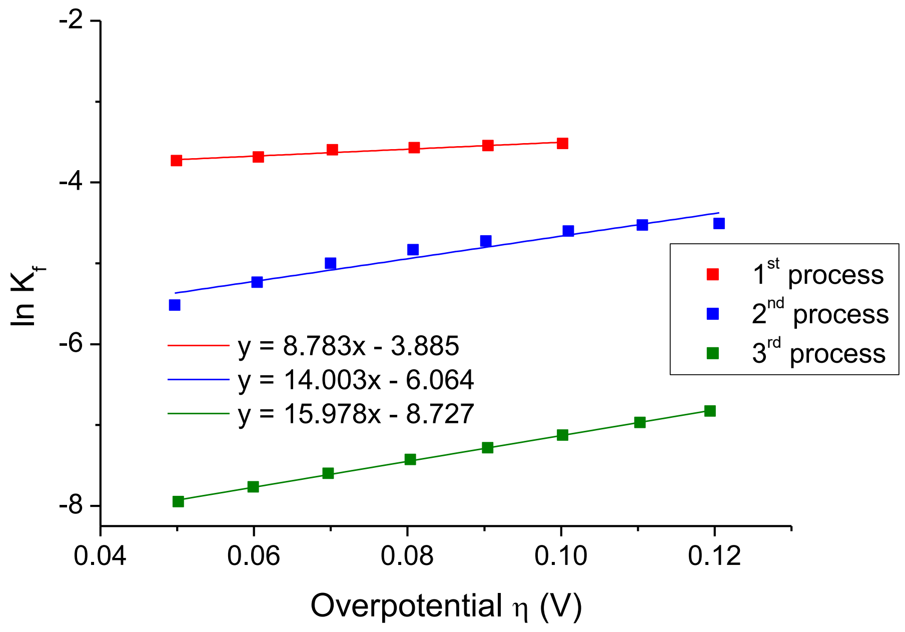

Figure S12: Tafel plot, the logarithm of kinetically limited current vs overpotential for the first (red) second (blue) and third (green) redox processes of 1mM of **(SPr)34TpyTz** in 1 M KCl.

3. Solubility. UV-Vis

By multiplying the concentration achieved from the sample interpolation (red crosses correspond to diluted 120000 times in Figure S13) by the corresponding dilution factor, the maximum solubility of the compounds was calculated.

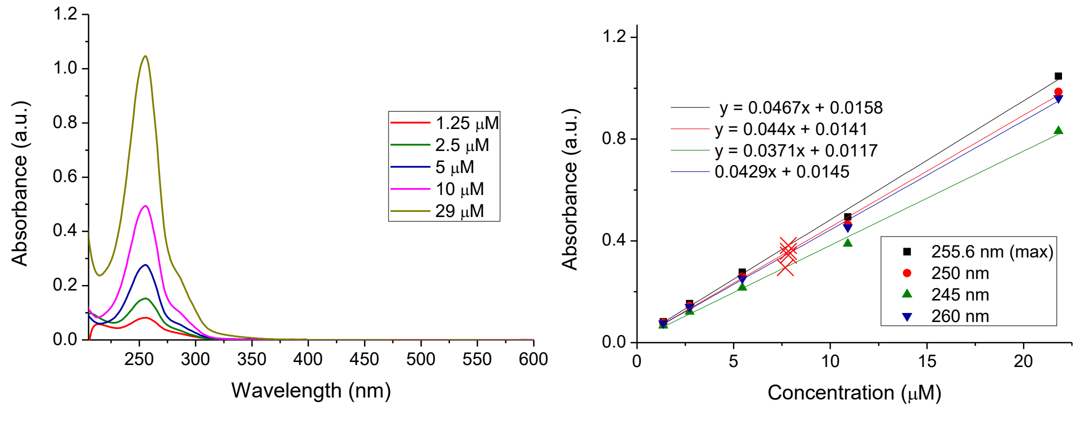

Figure S13: (a) UV-Vis spectra at different concentrations of compound **(SPr)34TpyTz** in 1 M KCl (b) Calibration curves at different wavelengths and saturated solution (red crosses).

4. DFT calculation, optimized geometries

**(SPr)$_3$4TpyTz**							
Oxidized				Reduced			
6	1.288148000	-0.223624000	-1.057641000	6	1.044190000	0.781271000	-1.374753000
6	-0.450522000	1.205076000	-1.061185000	6	-1.192470000	0.530528000	-1.367395000
6	-0.818679000	-1.014898000	-1.043739000	6	0.146036000	-1.306974000	-1.396272000
6	-0.972714000	2.594199000	-1.055451000	6	-2.554086000	1.126529000	-1.360936000
6	-2.345765000	2.831935000	-1.025335000	6	-3.686749000	0.313667000	-1.366220000
6	-0.100737000	3.676862000	-1.073004000	6	-2.726046000	2.509681000	-1.353906000
6	-2.801624000	4.128099000	-1.008932000	6	-4.934365000	0.888582000	-1.368319000
1	-3.056284000	2.017325000	-1.013745000	1	-3.595049000	-0.763241000	-1.371047000
6	-0.609525000	4.957791000	-1.052034000	6	-3.996947000	3.035316000	-1.356120000
1	0.970418000	3.533162000	-1.096827000	1	-1.873951000	3.174339000	-1.348905000
1	-3.854474000	4.377500000	-0.983811000	1	-5.848267000	0.309476000	-1.371964000
1	0.012001000	5.844519000	-1.038586000	1	-4.192397000	4.099735000	-1.350789000
6	2.751456000	-0.469265000	-1.056264000	6	2.238119000	1.666554000	-1.372027000
6	3.648331000	0.597626000	-1.059622000	6	2.093541000	3.051714000	-1.320453000
6	3.247292000	-1.767909000	-1.047185000	6	3.524808000	1.131571000	-1.423999000
6	4.997937000	0.337781000	-1.054677000	6	3.212243000	3.851200000	-1.323800000
1	3.303235000	1.621915000	-1.067942000	1	1.113640000	3.505343000	-1.278972000
6	4.609869000	-1.973963000	-1.039291000	6	4.609964000	1.974276000	-1.426392000
1	2.583403000	-2.621062000	-1.043938000	1	3.678879000	0.062569000	-1.464648000
1	5.744155000	1.121768000	-1.059563000	1	3.162378000	4.931440000	-1.283053000
1	5.063324000	-2.956972000	-1.008804000	1	5.631214000	1.618623000	-1.465590000
6	-1.759873000	-2.161747000	-1.025350000	6	0.301618000	-2.716648000	-1.411801000
6	-3.132586000	-1.950965000	-1.087775000	6	-0.819229000	-3.594864000	-1.416962000
6	-1.279198000	-3.466991000	-0.937825000	6	1.585300000	-3.332958000	-1.421590000
6	-3.986863000	-3.032279000	-1.060188000	6	-0.642239000	-4.941772000	-1.432900000
1	-3.544192000	-0.953549000	-1.153030000	1	-1.824503000	-3.197422000	-1.408578000
6	-2.173130000	-4.510219000	-0.917185000	6	1.702359000	-4.686814000	-1.437360000
1	-0.219375000	-3.673541000	-0.887368000	1	2.481488000	-2.728457000	-1.416593000
1	-5.065638000	-2.939458000	-1.083009000	1	-1.469731000	-5.639811000	-1.434303000
1	-1.863875000	-5.544752000	-0.850668000	1	2.660708000	-5.190145000	-1.441131000
7	-1.334829000	0.211279000	-1.054377000	7	-1.113285000	-0.779162000	-1.385581000
7	0.483925000	-1.283407000	-1.043838000	7	1.257703000	-0.513588000	-1.393972000
7	0.869731000	1.038952000	-1.065706000	7	-0.156254000	1.387603000	-1.358381000
7	-3.499282000	-4.282781000	-0.983666000	7	0.604440000	-5.498664000	-1.458725000
7	5.455983000	-0.929453000	-1.048754000	7	4.442645000	3.310263000	-1.384621000
7	-1.937917000	5.161905000	-1.026912000	7	-5.073868000	2.228453000	-1.371699000
6	-4.422148000	-5.442636000	-0.896403000	6	0.760005000	-6.952557000	-1.354419000
1	-5.351255000	-5.145260000	-1.377723000	1	-0.077198000	-7.414252000	-1.880964000
1	-3.967062000	-6.234633000	-1.488346000	1	1.678572000	-7.226696000	-1.876021000
6	-4.636781000	-5.895083000	0.557285000	6	0.803380000	-7.403340000	0.098924000
1	-3.912839000	-5.398505000	1.207354000	1	-0.110846000	-7.082147000	0.606002000
1	-4.424229000	-6.964180000	0.617824000	1	1.648627000	-6.925224000	0.602210000
6	-6.038945000	-5.688139000	1.115742000	6	0.936297000	-8.913627000	0.190558000
1	-6.792407000	-6.216280000	0.526642000	1	0.084472000	-9.416823000	-0.273851000
1	-6.078937000	-6.070863000	2.137410000	1	1.847931000	-9.265013000	-0.299324000
6	-2.478962000	6.541035000	-0.939713000	6	-6.428897000	2.818024000	-1.306510000
1	-3.367560000	6.557124000	-1.567975000	1	-7.099402000	2.141938000	-1.836751000
1	-1.734868000	7.201623000	-1.379263000	1	-6.395850000	3.765881000	-1.843445000
6	-2.816349000	6.926365000	0.510099000	6	-6.861525000	3.010865000	0.138241000
1	-3.852113000	7.269824000	0.542160000	1	-6.840922000	2.048881000	0.657593000
1	-2.764718000	6.040787000	1.147597000	1	-6.160723000	3.681433000	0.643166000
6	-1.962778000	8.033779000	1.114367000	6	-8.263206000	3.594035000	0.189242000
1	-2.307992000	8.243299000	2.128732000	1	-8.988751000	2.925703000	-0.280826000
1	-2.031778000	8.957928000	0.535833000	1	-8.311557000	4.561151000	-0.317186000
6	6.922586000	-1.154170000	-0.988915000	6	5.629806000	4.189287000	-1.315124000
1	7.111844000	-2.135426000	-1.419066000	1	6.417487000	3.714629000	-1.900225000
1	7.368896000	-0.403892000	-1.638901000	1	5.362431000	5.131727000	-1.792839000
6	7.464181000	-1.033409000	0.445188000	6	6.056579000	4.396537000	0.129386000
1	8.296955000	-0.327455000	0.436522000	1	5.234786000	4.847568000	0.692208000
1	6.698409000	-0.600204000	1.092743000	1	6.284792000	3.428714000	0.584253000
6	7.983576000	-2.322087000	1.070574000	6	7.279615000	5.295352000	0.190910000
1	8.363135000	-2.107653000	2.071450000	1	7.070089000	6.283375000	-0.226249000
1	8.798508000	-2.755289000	0.485843000	1	8.119134000	4.863029000	-0.359045000
16	6.764464000	-3.622564000	1.276477000	16	7.849238000	5.555927000	1.869002000
16	-0.213432000	7.659521000	1.256602000	16	-8.829048000	3.866009000	1.866729000
16	-6.578367000	-3.979347000	1.212245000	16	1.010980000	-9.492542000	1.883462000
8	0.333671000	8.681601000	2.182069000	8	-10.203438000	4.412858000	1.728131000

8	0.352383000	7.777027000	-0.119649000	8	-7.876374000	4.835681000	2.469695000
8	5.546443000	-2.967300000	1.823236000	8	6.717348000	6.191924000	2.593874000
8	7.379935000	-4.596952000	2.210367000	8	9.032093000	6.446649000	1.743907000
8	-6.917658000	-3.563968000	-0.180828000	8	-0.250070000	-9.040087000	2.529175000
8	-5.429885000	-3.213020000	1.765101000	8	2.220537000	-8.864760000	2.479609000
8	-7.765482000	-3.989549000	2.102339000	8	1.113607000	-10.972489000	1.789057000
8	-0.123473000	6.272675000	1.786345000	8	-8.796370000	2.537048000	2.532083000
8	6.535595000	-4.202266000	-0.079880000	8	8.189605000	4.208978000	2.399546000

5. Cell testing

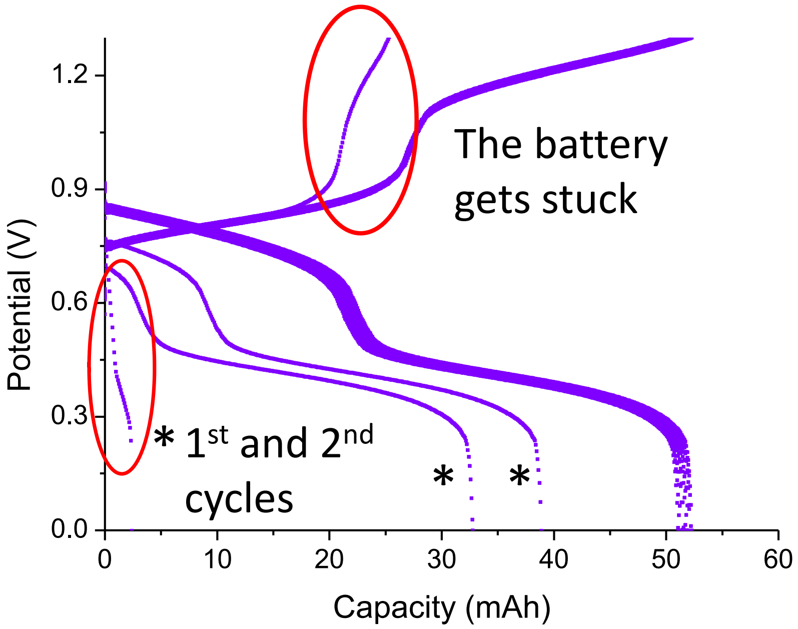

Figure S14: (a) E_{cell} vs Capacity plot at 60 mA/cm^2 for the battery (which get stuck in the 15th cycle) using 100 mM of **(SPr)$_3$4TpyTz** in 1 M KCl vs 300 mM $K_4[Fe(CN)_6]$ in 1 M KCl as electrolytes and N212$^{®}$ membrane.

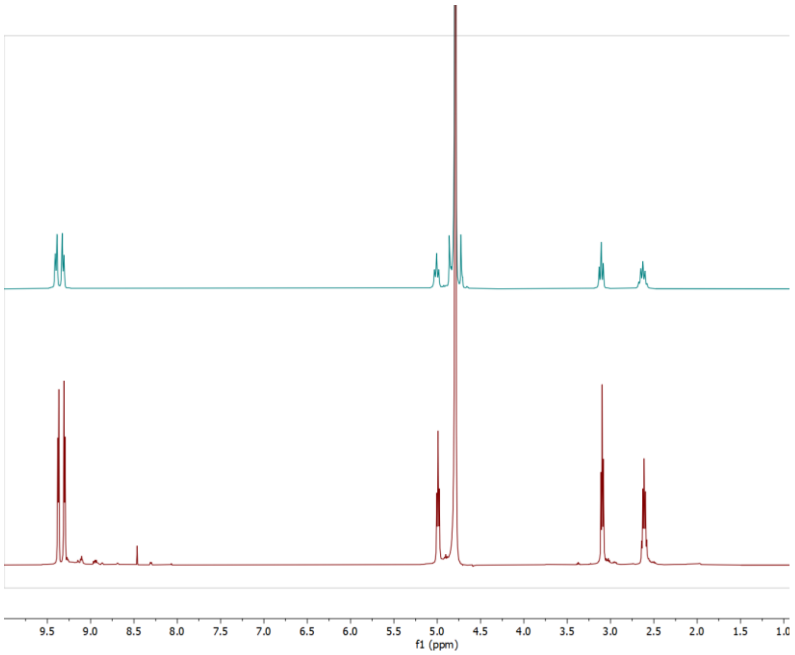

Figure S15: ^{1}H NMR spectra of anolyte 100 mM **(SPr)$_3$4TpyTz** in 1M KCl after the battery gets stuck, measured in D_2O before (blue trace) and after stucking (red trace). The peaks with small intensity in the red spectra could be ascribed to the reduced form of the **(SPr)$_3$4TpyTz.**

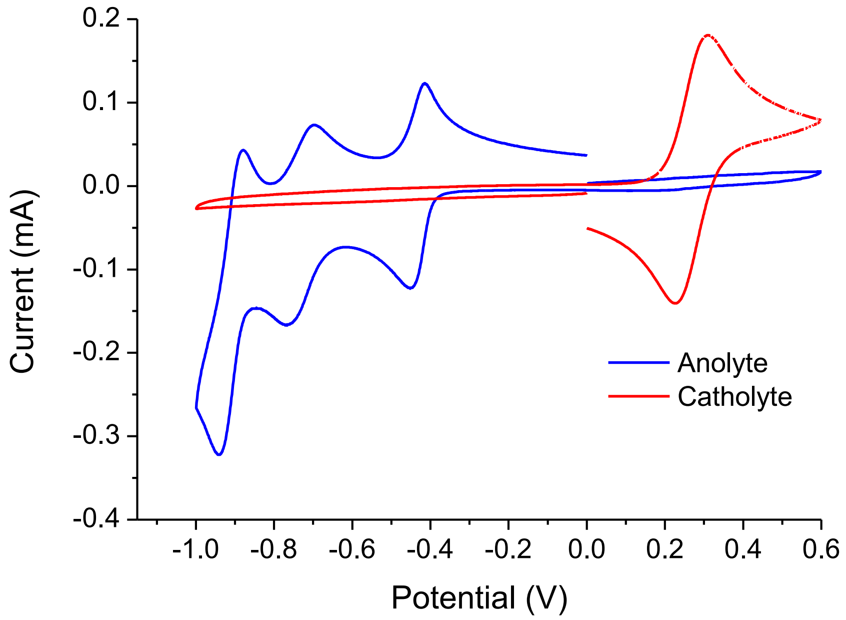

Figure S16: CV of anolyte 100 mM **(SPr)$_3$4TpyTz** in 1M KCl and catholyte 100 mM $K_4[Fe(CN)_6]$ in 1 M of KCl after the battery gets stuck.

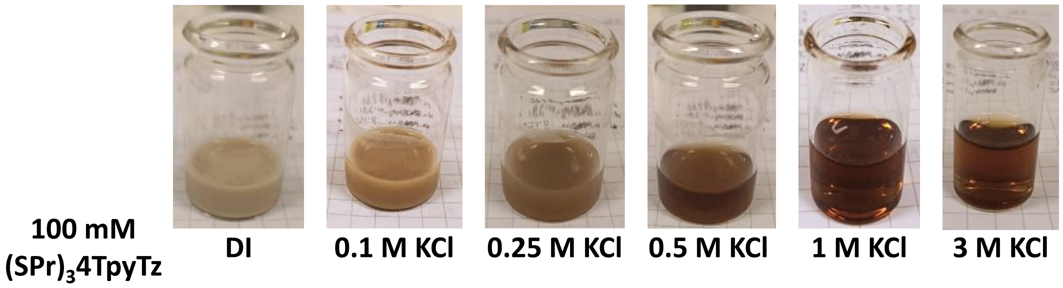

Figure S17: Pictures of the solution 100 mM of **(SPr)$_3$4TpyTz** ~~at~~ with different concentrations of KCl.

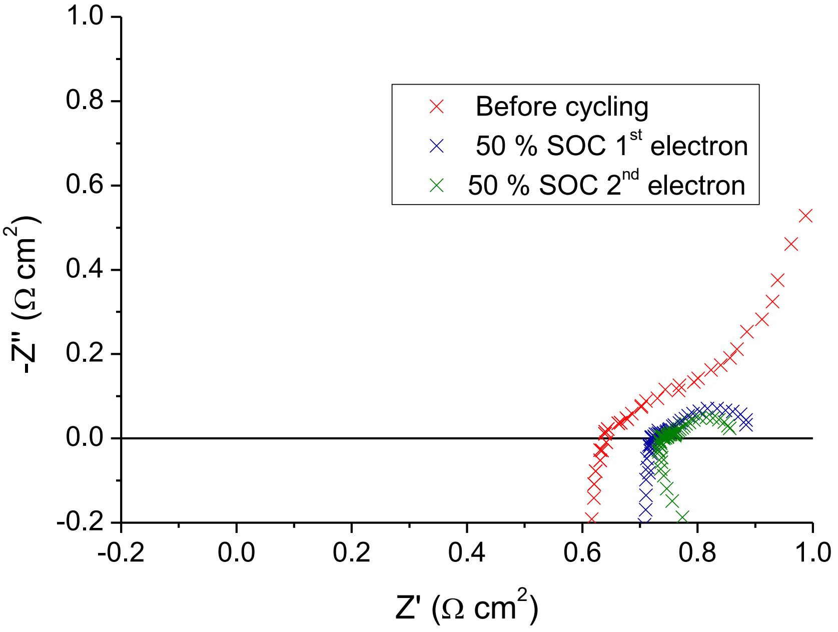

Figure S18: Electrochemical impedance spectroscopy for the battery 100 mM **(SPr)$_3$4TpyTz** in 3M KCl vs 100 mM $K_4[Fe(CN)_6]$ in 1 M of KCl before cycling (red crosses) 50 % SOC for the first process (blue crosses) and 50 % SOC for the second process (green crosses).

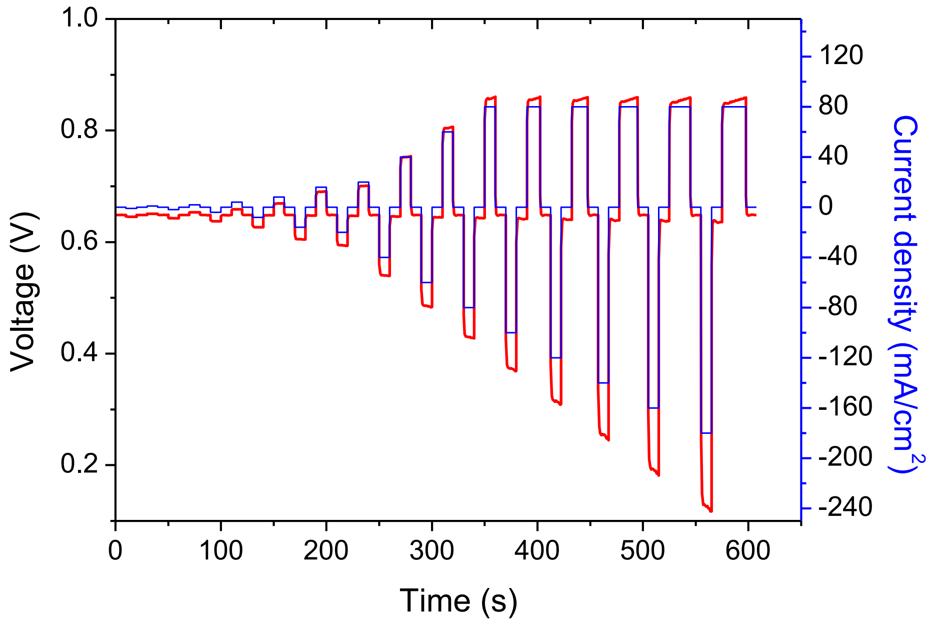

Figure S19: Current and voltage profile at consecutive charging, OCV and discharging at different currents for polarization curve of the first electron of the battery 100 mM **(SPr)$_3$4TpyTz** in 3M KCl vs 100 mM $K_4[Fe(CN)_6]$ in 1 M of KCl.

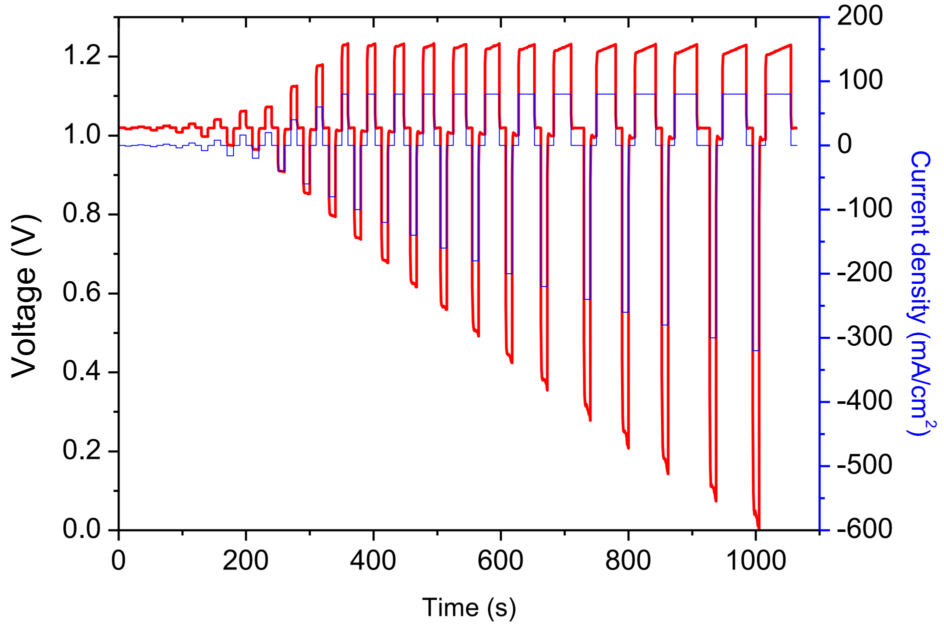

Figure S20: Current and voltage profile at consecutive charging, OCV and discharging at different currents for polarization curve of the second electron of the battery 100 mM **(SPr)$_3$4TpyTz** in 3M KCl vs 100 mM $K_4[Fe(CN)_6]$ in 1 M of KCl.

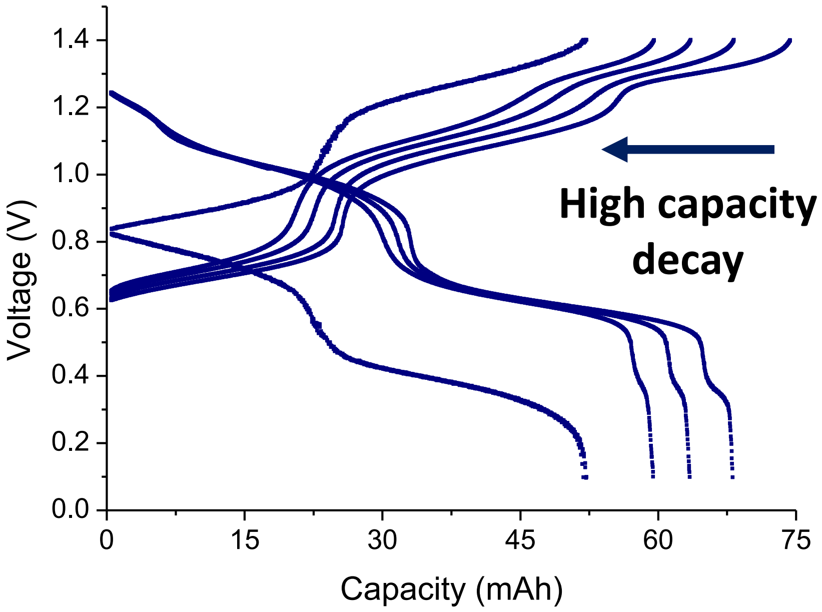

Figure S21: E_{cell} vs Capacity plot at 60 mA/cm^2 for the battery 100 mM **(SPr)$_3$4TpyTz** in 3M KCl vs 100 mM $K_4[Fe(CN)_6]$ in 1 M of KCl considering the third process.

Table S1: Comparison of the initial pH and the pH after reaching the third redox process for the battery 100 mM **(SPr)$_3$4TpyTz** in 3M KCl vs 100 mM $K_4[Fe(CN)_6]$ in 1 M of KCl:

	pH initial	*pH after 3rd process*
Anolyte	3.8	12.22
Catholyte	8.0	10.22

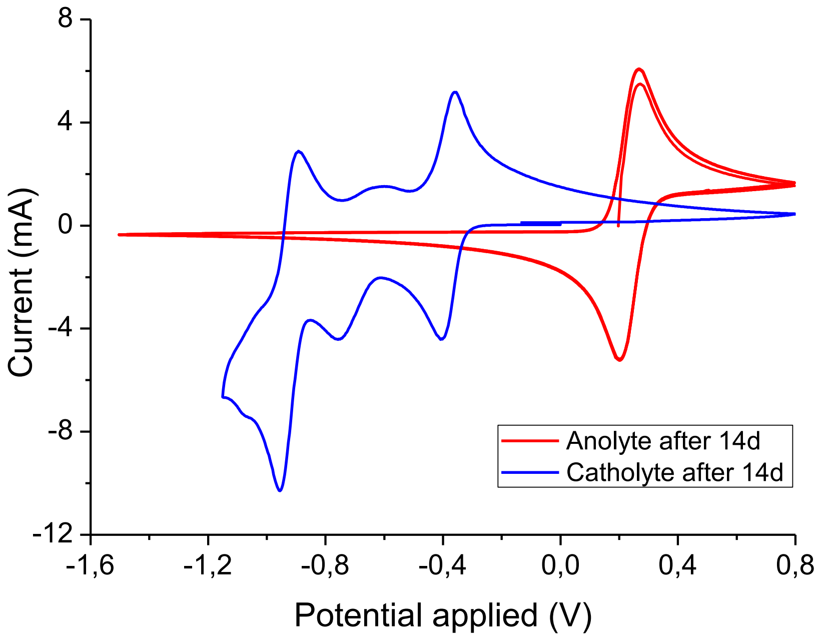

Figure S22: CV of anolyte 200 mM **(SPr)$_3$4TpyTz** in 3M KCl and catholyte 300 mM $K_4[Fe(CN)_6]$ in 1 M of KCl after 14 days of battery cycling.

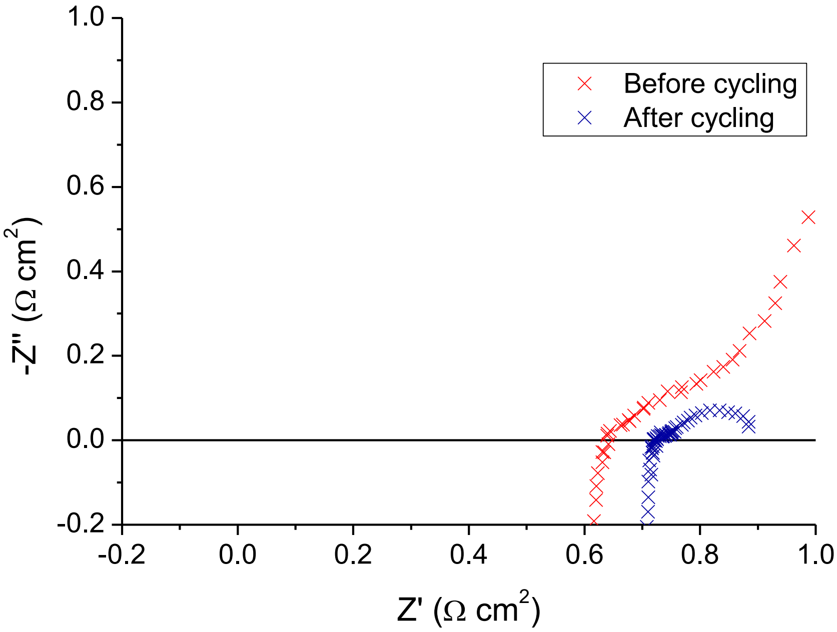

Figure S23: Electrochemical impedance spectroscopy for the battery 200 mM **(SPr)$_3$4TpyTz** in 3M KCl vs 300 mM $K_4[Fe(CN)_6]$ in 1 M of KCl before and after 14 days of cycling the battery.

6. Comparison with other organic electrolytes

In the following table S2, a brief coparison between the **$(SPr)_3$4TpyTz** with other viologen and triazine is depicted. Since **$(SPr)_3$4TpyTz** represent a sulfonated triazine it seems logical to compare our results with other similar organic electrolytes. Some of the examples here showed used different strategies to enhance their performance such as the use of symmetrical electrolytes or the use of a potentiostatic holding at the end of the galvanostatic cycling. Analyzing the results of the sulfonated compound it seems clear that the use of symmetric electrolytes enhances the solubility. Otherwise, the compound R-Vi has been just studied using 0.1 M concentration.

**Anolyte**	**Catholyte**	**Cycles**	**Capacity retention (%)**	**EE (%)**	**current density (mA/cm^2)**	**Ref**
$(CBu)_2V$ 0.85 M + $(NH_4)_4[Fe(CN)_6]$ 0.85 M	$(CBu)_2V$ 0.85 M + $(NH_4)_4[Fe(CN)_6]$ 0.85 M	1000	100	59.8	40	2
$(SPr)_2V$ 0.9 M + $(NH_4)_4[Fe(CN)_6]$ 0.9 M	$(SPr)_2V$ 0.9 M + $(NH_4)_4[Fe(CN)_6]$ 0.9 M	1000	100	62.6	40	3
3,4-S_2V 1.1 M + $(NH_4)_4[Fe(CN)_6]$ 1.1 M	3,4-S_2V 1.1 M + $(NH_4)_4[Fe(CN)_6]$ 1.1 M	1700	100	66.0	40	4
BTMAP-Vi 0.75M	BTMAP-Fc 1,0 M	500	99.45	n.s	50**	5
BPP-Vi 1 M<br>14 M NH_4OH	$K_{3.5}[Fe(CN)_6]$ 0.3 M<br>2M NH_4Cl	290	99.79	66.3	100**	6
R-Vi 0.1M<br>1 M KCl	$K_4[Fe(CN)_6]$ 0.1<br>1 M KCl	3200	77.6	87,0	50**	7
Dex-Vi<br>(1.5 M)	BTMAP-Fc<br>0.75 M	350	100	n.s	50**	8
$(TPyTz)Cl_6$<br>0.3 M (x 3 electrons)	FcNCl<br>n. s.*	50	n.s	n.s	80	9
**$(SPr)_3$4TpyTz 0.1 M (x 2 electrons) 3 M KCl**	$K_4[Fe(CN)_6]$ 0.1<br>1 M KCl	180	100	66.5	60	This work
**$(SPr)_3$4TpyTz 0.2 M (x 2 electrons) 3 M KCl**	$K_4[Fe(CN)_6]$ 0.3<br>1 M KCl	500	93.8	55.3	60	This work

* n. s.: not specified

**Potentiostatic holding

References

[1] Kwabi, D. G.; Ji, Y.; Aziz, M. J. Electrolyte Lifetime in Aqueous Organic Redox Flow Batteries: A Critical Review. *Chem. Rev.* **2020**, *120*, 6467-6489.

[2] Wu, W.; Wang, A.P.; Luo, J.; Liu, T. L. A Highly Stable, Capacity Dense Carboxylate Viologen Anolyte towards Long-Duration Energy Storage. *Angew. Chem. Int. Ed.* **2023**, *62*, e202216662.

[3] Luo, J.; Hu, B.; Debruler, C.; Bi, Y.; Zhao, Y.; Yuan, B.; Hu, M.; Wu, W.; Liu, T. L. Unprecedented Capacity and Stability of Ammonium Ferrocyanide Catholyte in pH Neutral Aqueous Redox Flow Batteries. *Joule*. **2019**, *3*, 149-163.

[4] Hu, M.; Wu, W.; Luo, J.; Liu, T. L. Desymmetrization of Viologen Anolytes Empowering Energy Dense, Ultra Stable Flow Batteries toward Long-Duration Energy Storage. *Adv. Energy Mater.* **2022**, *12*, 2202085-2202093.

[5] Beh, E. S.; De Porcellinis, D.; Gracia, R. L.; Xia, K. T.; Gordon, R. G.; Aziz, M. J. A Neutral pH Aqueous Organic–Organometallic Redox Flow Battery with Extremely High Capacity Retention. *ACS Energy Lett.* **2017**, *2*, 639-644.

[6] Jin. S.; Fell, E. M.; Vina-Lopez, L.; Jing, Y.; Michalak, P. W.; Gordo, R. G.; Aziz, M. J. Near Neutral pH Redox Flow Battery with Low Permeability and Long-Lifetime Phosphonated Viologen Active Species. *Adv. Energy Mater.* **2020**, *10*, 2000100-2000110.

[7] Li, H.; Fan, H.; Hu, B.; Hu, L.; Chang, G.; Song, J. Spatial Structure Regulation: A Rod-Shaped Viologen Enables Long Lifetime in Aqueous Redox Flow Batteries. *Angew. Chem. Int. Ed.* **2021**, *60*, 26971-26977.

[8] Lv, X.-L.; Sullivan, P.; Fu, H.-C.; Hu, X.; Liu, H.; Jin, S.; Li. W.; Feng, D. Dextrosil-Viologen: A Robust and Sustainable Anolyte for Aqueous Organic Redox Flow Batteries. *ACS Energy Lett.* **2022**, *7*, 2428-2434.

[9] Huang, J.; Hu, S.; Yuan, X.; Xiang, Z.; Huang, M.; Wan, K.; Piao, J.; Fu, Z.; Liang, Z. Radical Stabilization of a Tripyridinium–Triazine Molecule Enables Reversible Storage of Multiple Electrons. *Angew. Chem. Int. Ed.* **2021**, *60*, 20921-20925.
